# T1- vs. T2-based MRI measures of spinal cord volume in healthy subjects and patients with multiple sclerosis

**DOI:** 10.1186/s12883-015-0387-0

**Published:** 2015-07-31

**Authors:** Gloria Kim, Fariha Khalid, Vinit V. Oommen, Shahamat Tauhid, Renxin Chu, Mark A. Horsfield, Brian C. Healy, Rohit Bakshi

**Affiliations:** Departments of Neurology, Brigham and Women’s Hospital, Laboratory for Neuroimaging Research, Partners MS Center, Harvard Medical School, Boston, MA USA; Departments of Radiology, Brigham and Women’s Hospital, Laboratory for Neuroimaging Research, Partners MS Center, Harvard Medical School, Boston, MA USA; Department of Cardiovascular Sciences, University of Leicester, Leicester Royal Infirmary, Leicester, UK; Laboratory for Neuroimaging Research, One Brookline Place, Brookline, MA 02445 USA

**Keywords:** Multiple sclerosis, Spinal cord, MRI, Atrophy, Physical disability

## Abstract

**Background:**

The reliable and efficient measurement of spinal cord atrophy is of growing interest in monitoring disease progression in multiple sclerosis (MS).

**Methods:**

We compared T1- and T2-weighted MRI for measuring cervical spinal cord volume in 31 patients with MS and 18 age-matched controls (NC) from T1-weighted gradient recalled echo and T2-weighted fast spin-echo 1.5 T axial acquisitions. The two sequences were matched on slice thickness, signal averages and voxel size. An active surface software tool determined the normalized mean cervical cord cross-sectional area.

**Results:**

T1-derived cord areas were higher than T2 areas in the whole cohort (estimated mean difference = 7.03 mm^2^ (8.89 %); 95 % Confidence Interval (CI): 5.91, 8.14; *p* < 0.0001) and in both groups separately. There were trends for lower spinal cord areas in MS vs. NC with both sequences. For the T1 cord area, the mean difference was 3.7 mm^2^ (4.55 %) (95 % CI: −1.36, 8.78; *p* = 0.15). For the T2 cord area, the difference was larger [mean difference 4.9 mm^2^ (6.52 %) (95 % CI: −0.83, 10.67); *p* = 0.091]. The T1 and T2 cord areas showed similar weak to moderate correlations with measures of clinical status and T2 spinal cord lesion volume in the MS group. Superficial spinal cord T2 lesions had no apparent confounding effect on the outlining tool. The mean intra-rater and inter-rater coefficients of variation ranged from 0.27 to 0.91 % for T1- and 0.66 to 0.99 % for T2-derived cord areas.

**Conclusion:**

T2-weighted images may prove efficient for measuring cervical spinal cord atrophy in MS, with the added advantage of lesion detectability.

## Background

Brain and spinal cord lesions and atrophy are key MRI findings in the evaluation of patients with multiple sclerosis (MS). Many MRI segmentation methods have been developed to quantify spinal cord atrophy, including manual, semi-automated, and fully automated segmentation algorithms [1–10]. Manual outlining of axially acquired spinal cord images was initially used to monitor cord atrophy [6]. However, this lacked efficiency and precision. The use of edge-finding tools led to higher reproducibility by reducing user interactions [6–8]. In the past few years, the level of automation has increased in cord contouring algorithms. The active surface method [2] simply requires that the user marks the center of the cord on several vertebral levels of interest. This allows a rapid semi-automated segmentation by measuring the cord cross-sectional areas along the length of the extracted surface parameter [2]. Most recently, fully automated spinal cord segmentation methods have also been developed to produce reliable and accurate cord contouring [1, 10].

Spinal cord atrophy is most relevant to progressive forms of MS, i.e. primary progressive (PP) and secondary progressive (SP), in which it closely links to physical disability [2–6]. In most research settings, T1-weighted gradient recalled sequences are used to measure spinal cord volume. However, T2-weighted spin-echo sequences are more routinely used in MS clinics to assess lesions in addition to their potential role in measuring spinal cord volume. We compared two commonly-available sequences to measure cord volume: 1) T2-weighted fast spin-echo (FSE) sequences; 2) T1-weighted gradient recalled echo (GRE) sequences.

## Methods

Subjects’ characteristics are listed in Table 1. In the MS group, disability was assessed by the Expanded Disability Status Scale (EDSS) and timed 25-foot walk. Subjects underwent cervical spinal cord MRI on the same Philips 1.5 T scanner. For both sequences, axial images were obtained with a voxel size of 0.898 × 0.898 × 3 mm (no interslice gaps) with the same inferior-superior coverage and 2 signal averages. One hundred fifty to 200 axial slices were acquired on each subject to cover the whole spinal cord from the foramen magnum to the inferior extent of the conus medullaris. For the purposes of this study, only slices extending from the superior aspect of the C1 vertebral body to the inferior aspect of C7 were analyzed. The repetition (TR) and echo (TE) times (in milliseconds) [mean (range)] were, for the GRE sequence, TR = 520 (439–612) and TE = 4.26 (4.24-4.34). For the FSE sequence, TR = 9436 (8822–10659); TE = 100 in all subjects. All subjects gave informed consent for this study, which was approved by the local ethics committee of Partners Health Care. This human research was in compliance with the Helsinki Declaration (http://www.wma.net/en/30publications/10policies/b3/index.html).

**Table 1:** Subject characteristics

	Multiple sclerosis	Normal controls
Number	*N* = 31	*N* = 18
Age (years) (mean ± SD)	43.3 ± 8.7	43.6 ± 8.1
Women, number (%)	21 (68 %)	14 (78 %)
Disease category, number (%)		
Relapsing-remitting	26 (84 %)	-
Secondary progressive	4 (13 %)	-
Primary progressive	1 (3 %)	-
Disease duration^a^ (years) (mean ± SD)	9.0 ± 8.3	-
Expanded Disability Status Scale score (mean ± SD)	2.1 ± 2.1	-
Timed 25-foot walk (mean ± SD)	6.0 ± 5.2	-
T1 normalized cord area (mm^2^) (mean ± SD)	77.7 ± 10.6	81.4 ± 7.0
T2 normalized cord area (mm^2^) (mean ± SD)	70.2 ± 10.5	75.1 ± 9.0

*SD* standard deviation

^a^time since first symptoms

**Fig. 1:**
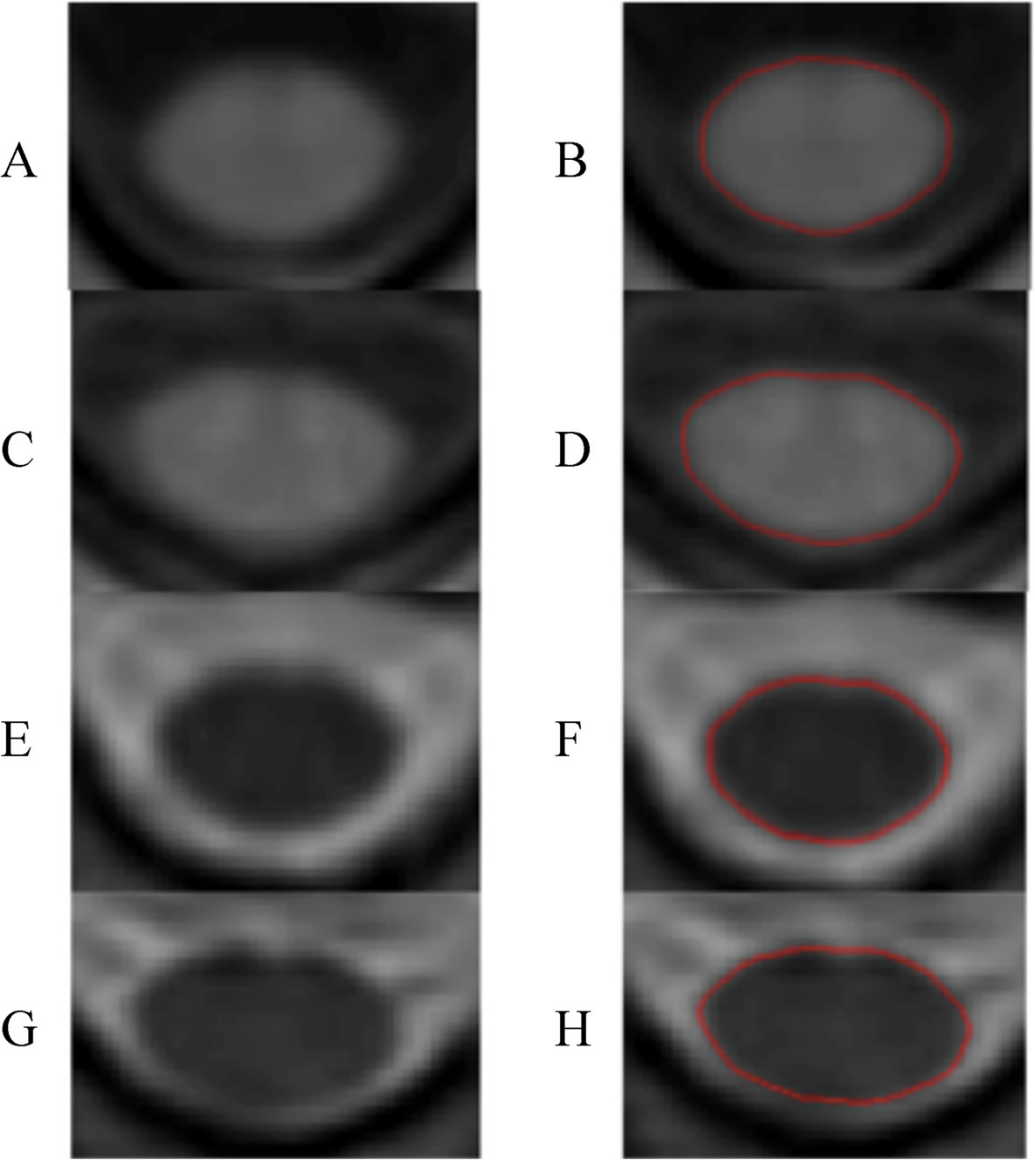
T1-weighted gradient echo (**a**–**d**) and T2-weighted fast spin-echo (e–h) axial MRI scans from C2. Left = raw images; right = cord outlines (*red*) after segmentation. **a**, **b**, **e**, **f**: Patient with relapsing-remitting MS [41 year-old woman, 5.4 years disease duration, low physical disability (EDSS score = 1)]. **c**, **d**, **g**, **h**: Normal control (49 year-old woman)

Image analysis was performed by a validated active surface method [2] using the Jim software package (v. 6.0 Xinapse Systems, West Bergholt, UK; www.xinapse.com). The whole cervical cord (C1 to C7) was measured (Fig. 1 shows sample images and segmentations). Spinal cord volume was normalized by dividing the total volume by the number of axial slices [3]. The operator analysis time per scan was the same for the T1 or T2 scans (~20 min). All MRI analysis was performed in a blinded manner, without knowledge of clinical details. To collect data on intra-rater and inter-rater reliability, five randomly chosen subjects (3 MS patients and 2 normal controls - NC) were analyzed. Two operators performed data analysis independently, with one rater analyzing the data a second time to assess intra-rater reliability.

**Fig. 2:**
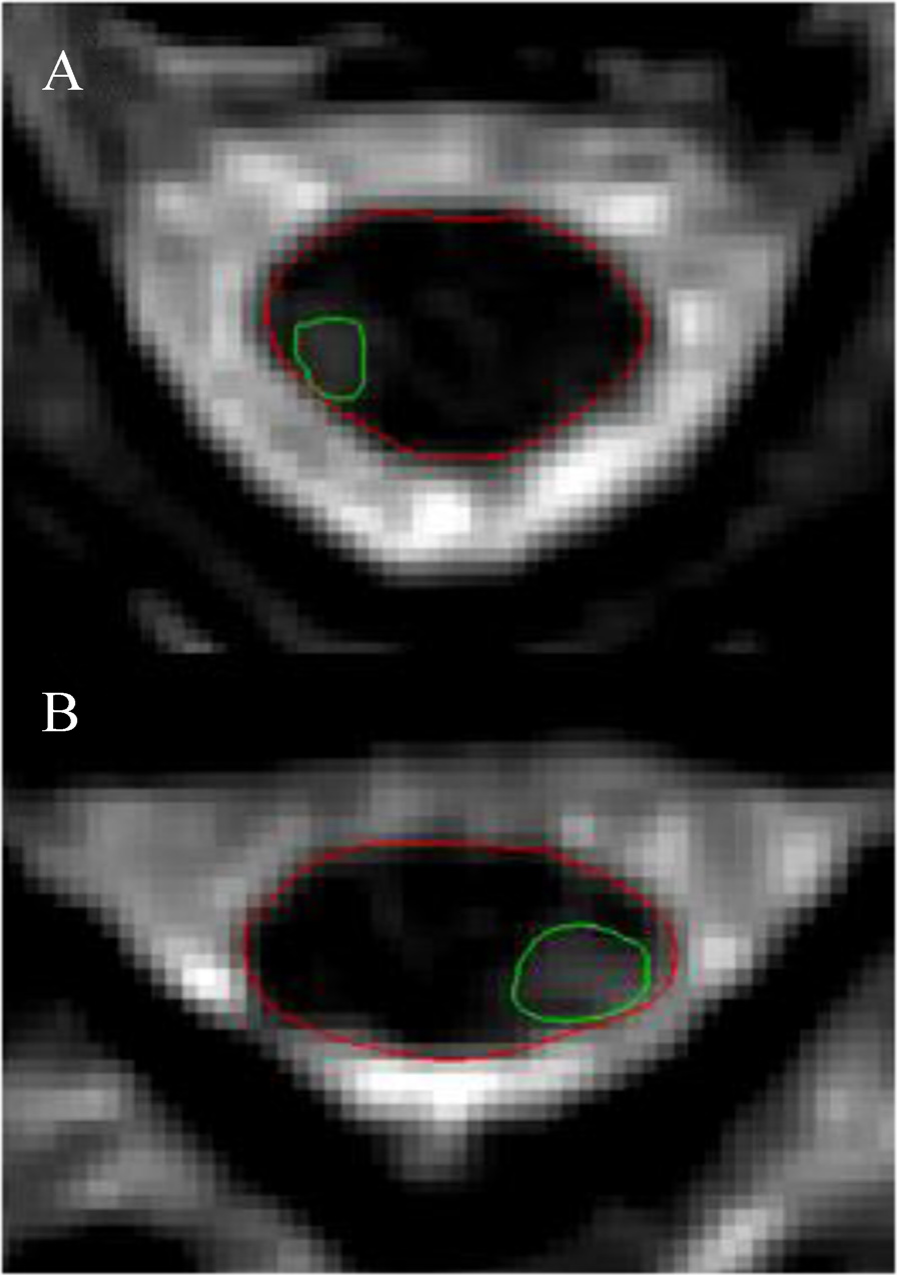
Performance of the cord-contouring tool with the presence of T2 hyperintense spinal cord lesions. Spinal cord lesion (*green*) and cord outline after segmentation (*red*). **a**: Axial slice at C2 from a patient with relapsing-remitting MS (52 year-old man, disease duration = 15.8 years, Expanded Disability Status Scale score [EDSS] = 1); **b**: Axial slice at C5 from a patient with relapsing-remitting MS (47 year-old man, disease duration = 12.4 years, EDSS = 0)

In addition, the presence of T2 hyperintense lesions in the cervical spinal cord was analyzed in all subjects to determine whether spinal cord lesions affected the cord contouring tool (Table 2, Fig. 2). The total volume and total number of cervical spinal cord T2 lesions were assessed. The number of lesions adjacent to the outer surface of the cord and the number of T2 lesions causing errors in the cord contour algorithm were also assessed. To confirm whether lesions in contact with the outer surface of the spinal cord interfered with cord segmentation, both the segmented cord outline and lesion tracings were overlaid using Jim software.

**Table 2:** T2 hyperintense cervical spinal cord lesions in the MS group

Patient #	Total cervical lesion volume (mm^3^)	Total # of cervical lesions	# of lesions contacting outer spinal cord surface	# of lesions causing contouring error
1	0	0	-	-
2	43.5	1	0	-
3	0	0	-	-
4	66.4	1	0	-
5	0	0	-	-
6	161.8	2	0	-
7	0	0	-	-
8	67.2	2	0	-
9	337.8	4	3	0
10	50.5	1	0	-
11	0	0	-	-
12	181.8	2	0	-
13	0	0	-	-
14	164.6	1	0	-
15	53.8	1	0	-
16	782.3	5	2	0
17	0	0	-	-
18	553.3	4	2	0
19	394.0	4	1	0
20	104.8	3	0	-
21	0	0	-	-
22	0	0	-	-
23	0	0	-	-
24	293.3	4	1	0
25	285.8	5	0	-
26	28.4	1	0	-
27	0	0	-	-
28	110.9	3	0	-
29	163.9	4	0	-
30	0	0	-	-
31	754.7	1	1	0
Mean	148.4	1.6	0.5	0
SD	215.7	1.7	0.9	0
Range	0–782.3	0–5	0–3	0-0

To assess agreement between T1 and T2 normalized cord areas, their within-subject differences were calculated and a paired *t*-test assessed significance. A Bland-Altman plot of the average normalized cord area vs. the difference between the two measures depicted the agreement between the two scans. Student’s t-tests compared groups. Spearman’s rank or Pearson’s correlations linked MRI to MRI, age or clinical measures. A p <0.05 was considered significant.

## Results

**Fig. 3:**
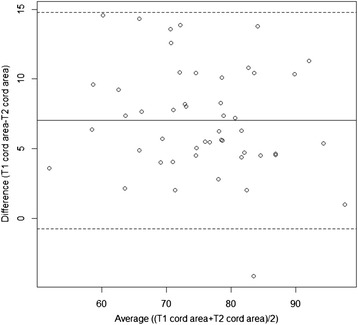
Bland-Altman plot showing all subjects – units of measure = mm^2^. T1 normalized cord areas are higher than T2 areas (estimated mean difference = 7.03 mm^2^; 95 % CI: 5.91, 8.14; *p* < 0.0001). Only one subject had a higher T2 normalized cord area than T1

Considering all subjects, the T1 cord areas were higher than the T2 areas [estimated mean difference = 7.03 mm^2^; 95 % Confidence Interval (CI): 5.91, 8.14; *p* < 0.0001, Table 1, Fig. 3]. This represented an 8.89 % difference [(mean difference/mean T1 cord area) × 100 %] between the mean T1 and T2 cord areas. This was consistent across all subjects (with only one NC having a higher T2 than T1 normalized cord area) and persisted when analyzing the two groups separately [estimated mean difference in MS = 7.47 mm^2^ (9.61 %); 95 % CI: 6.29, 8.65, *p* < 0.0001; NC = 6.26 mm^2^ (7.69 %); 95 % CI: 3.86, 8.67; *p* < 0.0001].

Data regarding the presence of spinal cord lesions on T2-weighted images are shown in Table 2 and Fig. 2. No lesions were present in the NC group. Among the 31 MS patients, 19 (61 %) had at least one spinal cord lesion. Six of these patients had lesions in contact with the outer spinal cord surface, none of which caused visually-apparent errors in the cord contouring algorithm (Fig. 2).

There were trends for lower spinal cord areas in the MS vs. NC groups when measured from the T1- or T2-weighted images (Table 1). For the T1 cord area, the mean difference was 3.7 mm^2^ (4.55 %) (95 % CI: −1.36, 8.78; *p* = 0.15). For the T2 cord area, the difference was larger, with MS showing greater atrophy, but this again did not reach significance [mean difference 4.9 mm^2^ (6.52 %) (95 % CI: −0.83, 10.67); *p* = 0.091]. The percent differences between MS and NC groups were calculated as the (mean difference/mean NC area) × 100 %.

The T1 and T2 cord areas showed similar weak to moderate correlations with three measures of clinical status in the MS group (Table 3). Spinal cord area vs. EDSS was similarly significant with both T1 (*p* = 0.032) and T2 (*p* = 0.033) cord measures (Table 3). Similarly, both T1 (*p* = 0.04) and T2 (*p* = 0.07) cord area measures showed approximately the same degree of negative association with the total volume of cervical spinal cord T2 lesions (Table 3).

**Table 3:** Correlation between spinal cord area and other variables in the MS group (*n* = 31)

	T1 normalized cord area		T2 normalized cord area	
	r_s_	*p*-value	r_s_	*p*-value
Age	−0.160	0.380	−0.200	0.290
Disease duration^a^	−0.249	0.177	−0.248	0.178
Expanded Disability Status Scale score	−0.386	0.032	−0.383	0.033
Timed 25-foot walk	−0.281	0.126	−0.260	0.157
Total cervical volume of spinal cord T2 lesions	−0.370	0.040	−0.330	0.070

*r*_*s*_Spearman coefficient

^a^years since first symptoms

Across all subjects, there were no correlations between age and T1 areas (*r* = −0.002, *p* = 0.99) or T2 areas (*r* = −0.001, *p* = 0.995). The MS group data are shown in Table 3. Considering just the NC group, there was a trend to correlation between age and T1 areas (*r* = 0.453, *p* = 0.059) and T2 areas (*r* = 0.419, *p* = 0.083); the interpretation of these trends was hampered by the small sample size in the NC group.

The method was highly reproducible, with mean coefficients of variation (COVs) of 0.27 % (intra-rater) and 0.91 % (inter-rater) for normalized cord area measured from T1 sequences and 0.66 % (intra-rater) and 0.99 % (inter-rater) from T2 sequences.

## Discussion

Despite differences in the volumes obtained, the two axial T1-weighted and T2-weighted sequences showed similar validity and reliability in the assessment of cervical spinal cord volume in MS. Regarding validity, the two measures showed similar differences between MS and NC and similar correlations with clinical measures in the MS group. Considering reliability, the intra-rater COV for each was acceptable.

A variety of acquisitions, including T1- and T2-weighted images can detect a relationship between spinal cord volume and MS disability [2, 5–10]. Spinal cord imaging is routine in MS care and provides unique and complementary information on disease severity that is not captured by brain MRI [8, 11, 12]. However, the “gold standard” images used to measure spinal cord volume in research settings (e.g. 3D T1-weighted, 1 mm isotropic voxels) are not typically feasible in routine care due to costs, scan time restrictions, and their questionable clinical applicability. Thus, clinically routine spinal cord images are more practical for large-scale MS studies. The T2-weighted FSE images have the added advantage of detecting inflammatory/demyelinating spinal cord lesions. Our results extend previous work [1, 2] and suggest that such images may provide a stand-alone tool to efficiently measure cervical spinal cord volume in MS.

In the present study, we showed no confounding effect of superficially placed T2 hyperintense spinal cord lesions on the accuracy of the cord contouring tool. However, it is important to note that we may not have seen an effect due to the fact that most of our MS patients were relapsing-remitting and the presence of spinal cord lesions was not ubiquitous. Future studies with larger sample sizes of progressive cases would be necessary to fully evaluate the potential confounding effect of lesions on the cord outline on T2-weighted images. Such progressive cohorts with more advanced disease will be required to fully evaluate the differences between MS and NC, which were not detected in our sample of mostly mildly disabled patients. While T2-weighted images showed high intra- and inter-rater reliability, the scan-rescan reliability remains to be determined. Longitudinal studies will uncover the sensitivity of T2-weighted images for monitoring spinal cord volume change and treatment effects. Further studies should also assess the role of 3D high-resolution scan acquisitions.

## Conclusion

T2-weighted images may prove efficient for measuring cervical spinal cord atrophy in MS, showing comparable effect sizes to T1-weighted images with the added advantage of lesion detectability.
